# Effects of Carbon Nanotubes on Mechanical Strength, Damage Process, and Microstructure of Lithium Tailing Backfilling

**DOI:** 10.3390/ma17163885

**Published:** 2024-08-06

**Authors:** Shufen Hu, Huadong Guan, Cai Wu, Yani Lu, Daopei Zhu

**Affiliations:** 1 School of Civil Engineering, Hubei Engineering University, Xiaogan 432000, China; hushufen86@gmail.com (S.H.); lyn2016@hbeu.edu.cn (Y.L.); 2 School of Civil and Surveying & Mapping Engineering, Jiangxi University of Science and Technology, Ganzhou 341000, China; 6720231526@mail.jxust.edu.cn; 3 Department of Civil Engineering, Kyushu University, Fukuoka 819-0395, Japan

**Keywords:** carbon nanotubes, cementitious backfills, compressive strength, hydration products

## Abstract

In this study, common multiwalled and carboxylated carbon nanotubes (CNTs) were added to the cemented lithium tailings backfill (CLTB). The effects of CNTs on the mechanical properties, hydration products, damage process, and microstructure of CLTB specimens were studied by uniaxial compression (UCS), infrared spectroscopy (FT-IR), and scanning electron microscopy (SEM). The experimental results show that the addition of CNTs effectively increased the compressive strength compared with the blank control group. When the concentration was 0.05–0.20%, the compressive strength was proportional to the content, the optimal addition amount was 0.2%, and the enhancement effect was 75% and 95.31%, respectively. The FT-IR results indicate that the addition of CNTs increased the total amount of the hydration product but did not affect its type. The hydration of the three-dimensional reciprocal penetration network formed by moderate amounts of CNTs has a positive effect on the mechanical strength of CLTB specimens.

## 1. Introduction

With the development of the economy, there has been a significant increase in demand for mineral products, leading to an expansion in the scale of mining operations. Metal mines generate a large amount of solid waste, such as tailings and waste residues, during the extraction process [1]. Additionally, the declining grade of many exploitable metal ores has necessitated larger-scale ore beneficiation to meet the growing demand for mineral products. Consequently, the amount of tailings generated from ore beneficiation processes is also increasing, posing numerous environmental challenges to the mining industry, the environment, and the economy [2,3,4]. Cemented tailings backfill (CTB) technology offers a solution for stabilizing and improving the efficiency of mining operations while reducing solid waste [5,6].

Evidence has shown that CTB technology not only controls ground pressure and large-scale strata movement in operating mines but also significantly improves the recovery rate and grade of ore, maximizing the extraction of valuable metals and high-grade ore [7,8]. Mining operations not only extract valuable ore but also result in significant underground voids. These voids pose a major safety hazard to mines due to pillar failure and roof collapse during low-pressure activities and strata movement [9]. To meet the increasing demand and requirements for green production, tailings cemented backfill technology is widely used in mines [10,11]. CTB is a composite material formed by mixing ore tailings with binders and water [12]. After preparation on the surface, CTB is sent into the mine voids through pipelines or pumps for backfilling. Cemented backfill is most commonly used for ground support in underground mines to control the ground pressure and large-scale strata movement in operating mines [13]. Therefore, the strength characteristics of backfill materials are crucial design considerations [14], and filling slump flow and compressibility are also important working indicators that determine the feasibility of cementing backfill [15,16,17]. In practical applications, CTB exhibits high brittleness and low ductility similar to those of concrete after hardening [18,19,20]. In recent years, an effective method for addressing brittleness and low ductility has been the application of fibers when preparing cemented backfill materials. Huang, Xue, and Jin reported that the addition of composite fibers to CTB promoted the development of post-peak toughness in specimens and prevented the propagation of large cracks [21,22,23,24]. Cao, in a series of studies, reported that the type, length, content, and shape of fibers all influence the enhancement effect [25]. Notably, fiber entanglement and balling impact can also affect the workability and destructiveness of the backfill [26,27]. Moreover, the production of synthetic fibers involves the consumption of a large amount of nonrenewable resources, and the nonbiodegradability of composite fibers increases the environmental burden, contradicting the principles of green mining.

Carbon nanotubes (CNTs), which are excellent nanoscale materials, have been found by Sarvandani, Alafogianni, Hawreen, Li, and others to effectively improve the mechanical strength and durability of cement mortar when trace amounts of CNTs are added to the cement matrix [28,29,30,31,32]. However, increasing the amount of CNT did not significantly enhance the strength. The addition of CNTs leads to nanoscale nucleation, filling, and bridging effects [33,34,35]. Barati Farimani mentioned in an article that the optimal concentration is 0.1% of the cement mass, and most researchers use multi-walled carbon nanotubes, with a few using functionalized modified CNTs [36]. Therefore, it is necessary to study the different effects of functionalized carbon nanotubes on the performance of cemented backfill materials. CNT-enhanced backfilling provides a new research direction for managing tailings and green mining technology [37,38].

This study aimed to investigate the influence of CNTPL and CNTCOOH mass concentrations (0.05–0.25%) on the performance of CTB. The enhancement effect of CNTs was primarily evaluated through uniaxial compression tests, with a control group of CTB without any additives at a binder-to-sand ratio of 1:8. The trend of the hydration products was observed using FTIR. The working mechanism of CNTs at the microscale will be examined through SEM.

## 2. Materials and Methods

### 2.1. Materials

#### 2.1.1. Tailings and Cement

**Figure 1:**
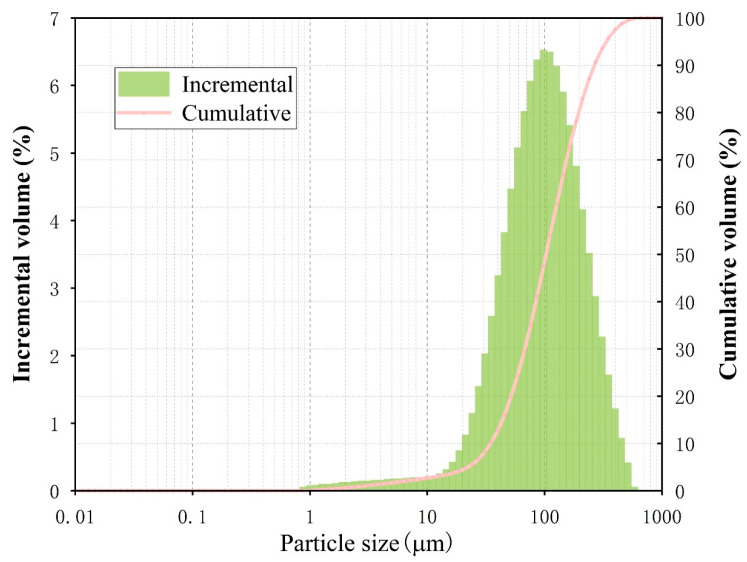
Tailings particle size.

As the primary material of the cemented filler, the raw material of this ore came from a company engaged in lithium mining and smelting in Yichun, Jiangxi Province, and the main component of the ore was lithium mica ore. The selected tailings were beneficiation tailings, which are solid mineral wastes produced in the sorting operation in the mine, and the tailings slurry formed the material through natural dewatering. Tailings, after constant temperature dehydration in an electric drying oven, underwent particle size analysis with a Malvern Mastersizer 3000 laser analyzer from Malvern Instruments Limited, Malvern, UK; Figure 1 presents the particle sizes of the tailings. The cement product was early-strength ordinary silicate cement (P.O 42.5R).

**Figure 2:**
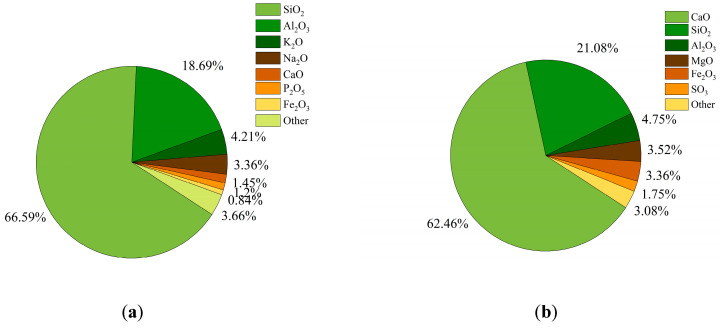
XRF spectral analysis: (**a**) tailings chemistry and (**b**) P.O 42.5R chemistry.

The chemical composition of the tailings cement was measured via a PANalytical Axios X-ray fluorescence (XRF) spectrometer from the Spectris plc, London, UK. Figure 2 illustrates the chemical compositions of the tailings and cement used in this study. Figure 2a shows that the tailings contained 66.5925% SiO_2_, 18.6931% Al_2_O_3_, and 1.4519% CaO, and Figure 2b shows the chemical composition of P.O 42.5R: 62.46% CaO, 21.08% SiO_2_, and 4.75% Al_2_O_3._

#### 2.1.2. Carbon Nanotube Characterization

Carbon nanotubes can be viewed as nanoscale tubular materials consisting of a hexagonal graphite sheet rotating around a central axis with closed or open ends. Depending on the number of layers, carbon nanotubes can be categorized as single, double, or multi-walled carbon nanotubes. Table 1 shows the physical parameters of carbon nanotubes.

As nanomaterials, CNTs have excellent mechanical properties, electrical conductivity, and heat transfer, even when they are used in low numbers. Compared with conventional fibers, multi-walled CNTs have great potential to improve material properties and mechanical properties. These materials had a modulus of elasticity of 1 TPa and a tensile strength of 50–200 GPa. Thus, scholars have found opportunities to use these materials frequently in various applications. Two types of CNTs were used in this study: common multiwall (CNTPL) and carboxylated (CNTCOOH) carbon nanotubes. CNTCOOH is CNTPL treated with acid to introduce a COOH functional group to increase the solubility and chemical reactivity while maintaining its excellent strength. The parameters of the two CNTs used are listed in Table 1.

### 2.2. Preparation of CLTB Specimens

The reinforcing effects of CNTs depend on their dispersion in solution. Due to the intermolecular forces resulting from the small size of CNTs, these tubes can quickly become entangled and tightly piled, increasing the difficulty of dispersion. In previous studies [36,39], it was found that if a single dispersion method was used to disperse CNTs, uniform dispersion could not be achieved, leading to agglomeration and stress concentration and affecting the reinforcing effect on the specimens. Therefore, in this paper, CNTs were treated using both chemical and physical methods. First, the hydrophilicity of the CNTs was increased by the surfactant polyvinylpyrrolidone (PVP), after which a blender was used to accelerate the fusion of the two materials and break up large agglomerates of CNTs to initially mix the CNTs with water. Then, ultrasonic treatment was used to break the strong van der Waals forces between the CNTs to form a homogeneous CNT suspension. The intensity, duration, and temperature of the ultrasonic dispersion affect the dispersion of CNTs. The best results were obtained after ultrasonic treatment at 60 °C for 30 min. To obtain a well-dispersed CNT suspension, the following procedure was used: first, the CNTs were mixed with tap water; second, a 1:1 mass of PVP was added; and finally, the mixture was stirred for 3 min to promote dissolution of the surfactant. Finally, this experiment used an ultrasonic cleaner to ultrasonicate the carbon nanotube solution at 60 °C for 30 min.

Moreover, Table 2 provides an overview of the material mix proportions of CLTB. The mass fraction of the experimentally prepared slurry was 70%, and the concentrations of CNTPL and CNTCOOH in the experimental group were 0.05%, 0.10%, 0.15%, 0.20%, and 0.25%, respectively, which are the mass fractions of water. As a control group, the cement/tailing ratios of the specimens without CNTs were 1:4, 1:6, and 1:8.

**Figure 3:**
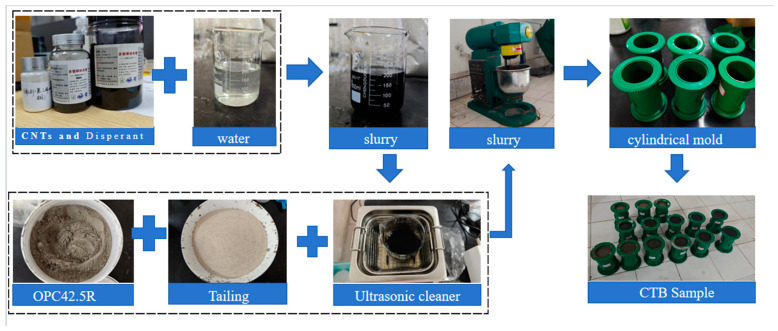
Critical fabrication steps for CLTB specimens.

CNTs and PVP were added in small amounts and weighed on electronic scales with an accuracy of 0.01 g; several materials, such as tailings, cement, and water, were weighed on ordinary electronic scales to an accuracy of 0.1 g. The tailings and cement were added to the mortar mixer and stirred for 1 min, and the aqueous solution of CNTs was added and stirred for 3 min. The prepared cementitious lithium tailings backfill (CLTB) slurry was poured into a cylindrical mold with a 50 mm diameter and 100 mm height (following the guidelines specified in the Chinese National Standard GB/T 39489-2020 [40]), which was first cured with the mold for one day and subsequently demolded. The specimens were cured at 21 °C/91% temperature/humidity for 7 days. Figure 3 shows the key steps in the preparation of the CNT-reinforced CLTB specimens.

### 2.3. Uniaxial Compressive Strength Tests

**Figure 4:**
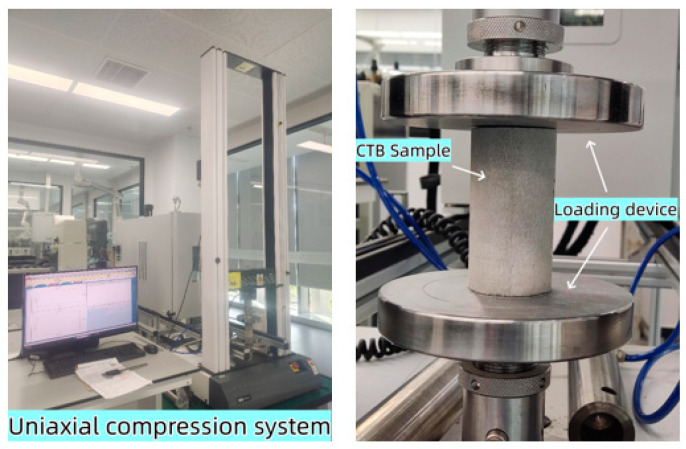
Electronic universal testing machine.

Uniaxial compressive tests were carried out after the CLTB specimens were cured for 7 days. An INSTRON 3343 electronic universal testing machine, from Instrong Test Equipment Trading Co., LTD., Shanghai, China (Figure 4) was selected for this experiment. The displacement speed control mode was 1 mm/min, and the test temperature was room temperature. During the stress loading process, the stress, deformation, and time data were recorded in real-time and plotted as stress variation curves. To avoid experimental errors, three specimens were tested in each set of tests, and the average value was taken as the final compressive strength. After the block strength reached 70% of the peak strength, it was considered block failure and 2.5% strain was set on the controller as the endpoint of the experiment.

### 2.4. FTIR Analysis

The fragments after the uniaxial compressive tests were taken, and the middle part of the fragments were used for detection. The chemical bonds of the CLTB specimen sample were characterized at the curing age of 7 days by means of FTIR spectroscopy (Nicolet iS 10, from Thermo Fisher scientific, Waltham, MA, USA). The wavenumber varied from 4000 cm^−1^ to 400 cm^−1^.

### 2.5. Scanning Electron Microscopy

**Figure 5:**
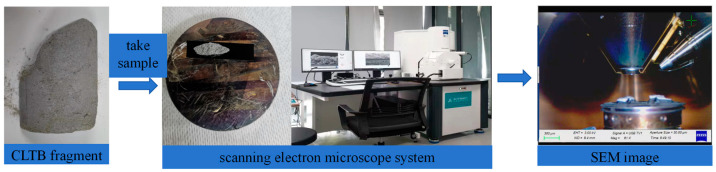
Electron microscopy test system.

After the uniaxial compression test, the specimens were subjected to CLTB in a sealed bag to save the broken specimens. Before observation, the former crushed CLTB specimens were dried and polished as needed, and the CLTB specimens were sprayed with gold to increase the conductivity of the samples to observe the clear morphologies of the hydration products and the distributions of the cracks. The scanning electron microscopy instrument used in this test was a German ZEISS Sigma 300 (From Zeiss in Jena, Germany), as shown in Figure 5.

## 3. Results and Discussion

### 3.1. Mechanical Properties of CNT-Reinforced CLTB Specimens

**Figure 6:**
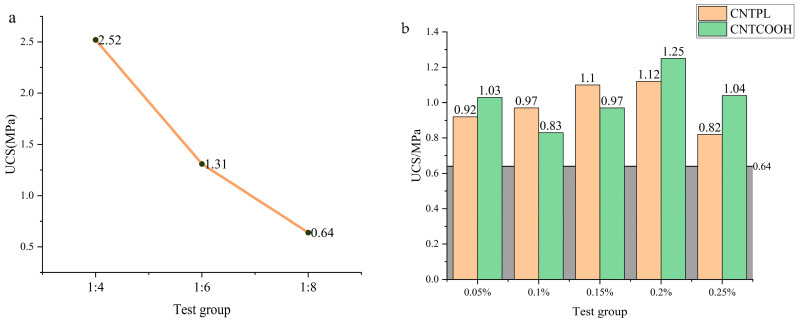
CLTB compressive strength: (**a**) ordinary CLTB specimen and (**b**) CNT-reinforced specimen.

Figure 6 shows line charts of the compressive strength of the ordinary CLTB and CNT-reinforced CLTB samples. Figure 6a shows that with an increasing cement/tailing ratio, the strength of the specimen decreases rapidly, and the strengths of the specimens are 2.52 MPa, 1.31 MPa, and 0.64 MPa. The cementitious material content is the key parameter affecting the compressive strength of the cemented filler in tailings, and the hydration products generated by cementitious materials are the main reason for the bonding between aggregates.

To explore the effect of the CNTs on strength, the growth rate was calculated as shown in Equation (1):

(1) $$f=\frac{f_{RF-CLTB}-f_{CLTB}}{f_{CLTB}}$$

where *f* is the growth rate of the UCS strength of the CLTB, $f_{RF-CLTB}$ is the compressive strength of the CLTB specimens reinforced with CNT, and $f_{CLTB}$ is the compressive strength of the CLTB with the cement/tailing ratios 1:8.

Figure 6b shows that when the cement/tailing ratio is 1:8, and the CNTPL content increases from 0.05% to 0.25%, the average UCS values are 0.92 MPa, 0.97 MPa, 1.10 MPa, 1.12 MPa, and 0.82 MPa. When the CNTCOOH content increased from 0.05% to 0.25%, the average UCS of the samples was 1.03 MPa, 0.83 MPa, 0.97 MPa, 1.25 MPa, and 1.04 MPa. The doping of CNTs can effectively improve the strength of CLTB specimens.

**Figure 7:**
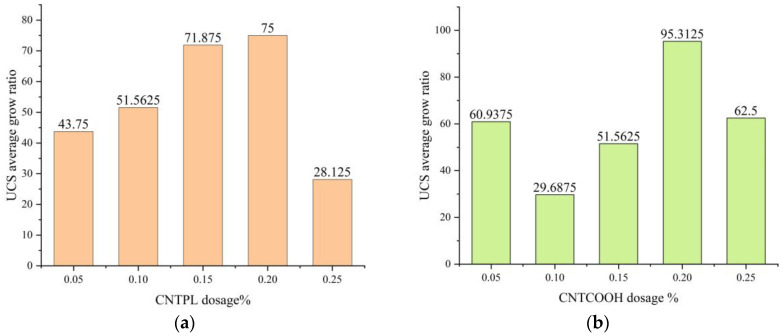
UCS growth rate of CLTB specimens. (**a**) The rate of increase of CNTPL; (**b**) The rate of increase of CNT.

Figure 7a shows that, compared with that of the CLTB specimen with a cement-tailing ratio of 1:8, the strength of the CLTB specimen with 0.05–0.25% added CNTPL significantly increased. The growth rates of intensity were 43.75%, 51.56%, 71.87%, 75%, and 28.125%, respectively.

Figure 7b shows that, compared with that of the ordinary CLTB specimens with a cement-tailings ratio of 1:8, the strength of the filled sample with a CNTCOOH content increased from 0.05% to 0.25%. The strength growth rates were 60.9%, 29.68%, 51.56%, 95.3%, and 62.5%. The strength of the CNTs first increased and then decreased.

The above results show that the addition of both CNT types significantly improved the compressive strength of the CLTB specimens, and the increase in compressive strength was optimal at 0.20%. The CNTPL enhancement was relatively stable, but the peak enhancement of CNTCOOH was higher.

### 3.2. Stress-Strain Properties of CNT-Reinforced Specimens and the CLTB Specimen Damage Process

**Figure 8:**
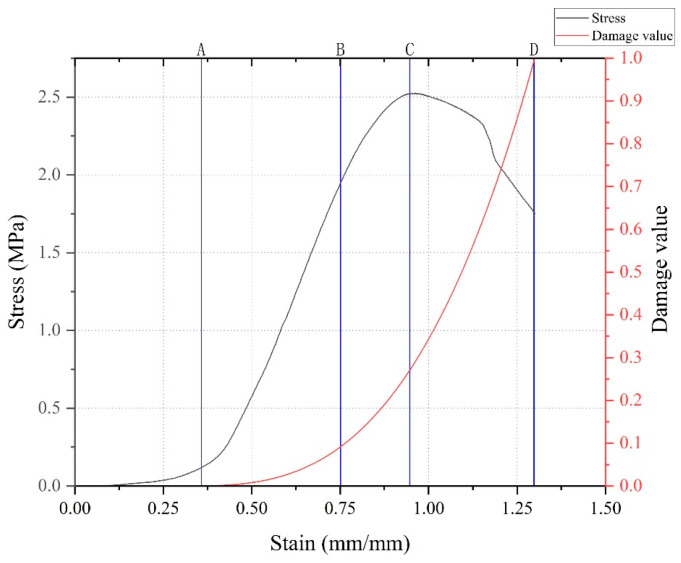
CLTB damage process and damage value of the sample.

**Figure 9:**
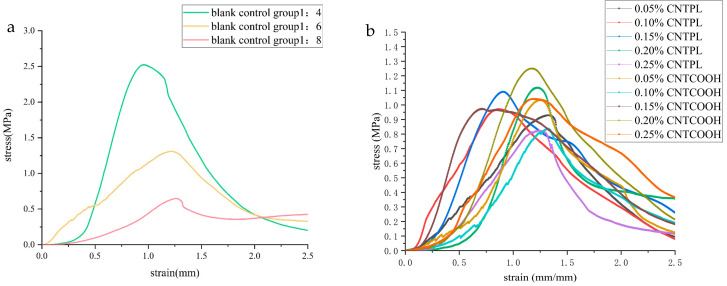
CLTB stress-strain curves for (**a**) blank control group and (**b**) CNT-reinforced specimens.

**Figure 10:**
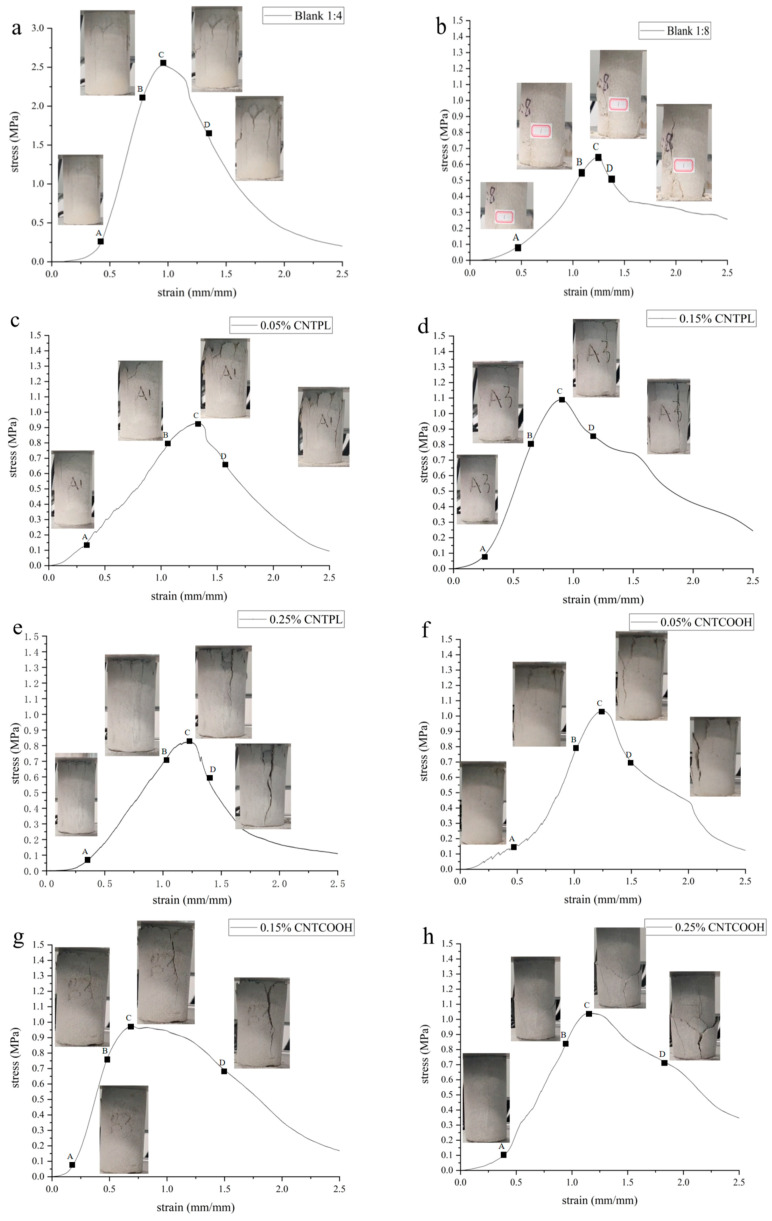
The main failure process of specimens (**a**) blank control 1:4, (**b**) blank control 1:8, (**c**) 0.05% CNTPL, (**d**) 0.15% CNTPL, (**e**) 0.25% CNTPL, (**f**) 0.05% CNTCOOH, (**g**) 0.15% CNTCOOH, (**h**) 0.25% CNTCOOH.

Taking the damage analysis of CLTB in the blank control group 1:4 in Figure 8 as an example, the stress-strain curves of the ordinary CLTB samples and CNT-reinforced CLTB samples in Figure 9 were analyzed together with the pictures recorded in the experiment and presented in Figure 10. The main injury process of CLTB and CNT-reinforced CLTB specimens is divided into four stages:

(1) In the pore compaction stage, there are certain pores and cracks in the cemented fill body, and the tiny pores inside the fill are gradually compacted under the action of loading. The pore compaction stage of the CLTB specimen containing carbon nanotubes is significantly shorter than that of the ordinary backfill specimen. There is no damage at this stage, and the curve at this stage is characterized by a concave shape.
(2) Damage stable development stage: During this process, the CLTB exhibits linear elastic deformation, indicating that the damage value of the test block begins to increase. The damage processes of the CLTB specimens with the addition of CNTs during this stage are consistent with those of the ordinary CLTB specimens. In this process, the damage value increases linearly, and subtle cracks can be observed on the surface.
(3) Damage acceleration stage: The load of the CLTB exceeds the elastic limit and changes from elastic deformation to plastic deformation. This stage occurs before the peak of the curve, at which point the CLTB specimen begins to break. The damage to the test block accelerates, the stress curve becomes convex, the inside of the sample begins to reach the stress limit, and the fine cracks observed in the second stage begin to accelerate, expand, and become obvious.
(4) Damage failure stage: In the post-peak failure phase, with increasing stress, the cracks in the CLTB specimen continue to expand and extend until the CLTB specimen is destroyed. The CLTB specimen is fully destroyed under uniaxial pressure, and obvious Y-type cracks and block detachments appear on the surface of the CLTB specimen.

A comparison of the curves and destruction processes of normal CLTB specimens and CNT-reinforced CLTB specimens in Figure 8, Figure 9 and Figure 10 shows that the addition of CNTs did not alter the injury process of CLTB. Both CLTB and CNT-reinforced CLTB show shear failure mode, the test block first appears in a direction perpendicular to the stress, and then the crack develops parallel to the stress, forming a crack through the test block. After the test, the X-type shear damage surface appeared inside the sample. However, the CNT-reinforced CLTB samples reached peak intensity earlier and generally had smaller slopes of the stress-strain curves when compared to the common CLTB samples. These results show that the nanofilling effect of CNTs reduces the time of the pore compaction phase and improves the rapid growth of the damage value of the test block.

By comparing the compressive strength of CLTB samples strengthened at different concentrations, it was found that with the increase of carbon nanotube concentration, the strengthening effect first increased and then decreased, for the following reasons:

(1) As mentioned in Section 2.2, the dispersion effect of CNTs affects the strength of the filler. A high concentration of 0.25% CNTs is difficult to completely disperse, and many CNTs are entangled and agglomerated, forming interfacial defects and stress concentrations in the CLTB specimen, which can be observed via electron microscopy analyses.
(2) The addition of the PVP activator produces many bubbles in aqueous solution, which input additional gases into the cementitious body during the mixing process, thereby increasing the porosity and decreasing the strength.

### 3.3. FTIR Analysis

**Figure 11:**
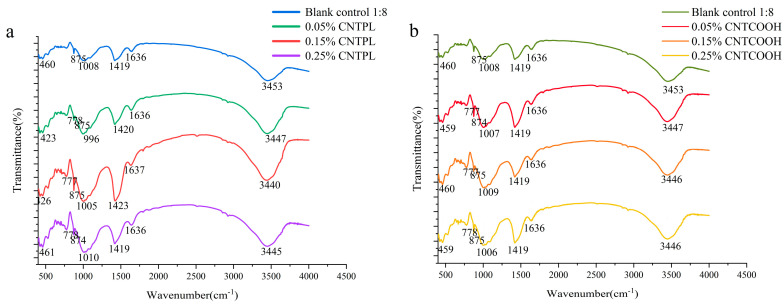
FT-IR pattern of specimens incorporated with different modified MWCNT numbers (**a**) compare with CNTPL (**b**) compare with CNYCOOH.

The FTIR spectra of CLTB samples are shown in Figure 11. The characteristic peaks of hydration products C-A-H and C-S-H are as follows: symmetrical vibration of O-Si-O (460 cm^−1^), Al-O bond (875 cm^−1^), asymmetric vibration of T-Si-O bond (1004 cm^−1^), and stretching vibration of O-H bond (3450 cm^−1^). The absorption peak near 1420 cm^−1^ is caused by the G-band peak of the carbon nanotubes and the symmetrical vibration of the O-C-O bond, and the acromion near 1636 cm^−1^ is caused by the presence of free water in the pore. The absorption peak near 777 cm^−1^ is caused by the vibration of the sulfur–oxygen bond and is a characteristic peak of hydrated calcium sulfoaluminate and monosulfate.

Figure 11a,b shows that the addition of CNTs significantly enhanced the absorption peaks at 3450 cm^−1^, 1420 cm^−1^, 777 cm^−1^, and 875 cm^−1^. In summary, the addition of CNTs did not change the type of FTIR absorption peak, but enhanced the strength of the characteristic peak of hydration products, indicating that the addition of CNTs played a role in nanonucleation and promoted the generation of hydration products.

### 3.4. Microstructures of CNT-Reinforced CLTB Specimens

**Figure 12:**
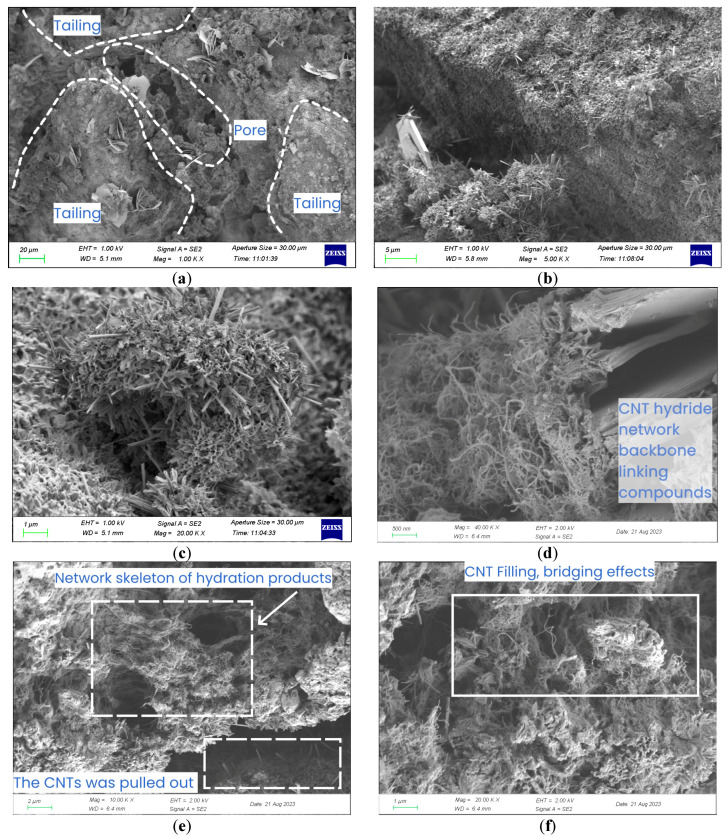

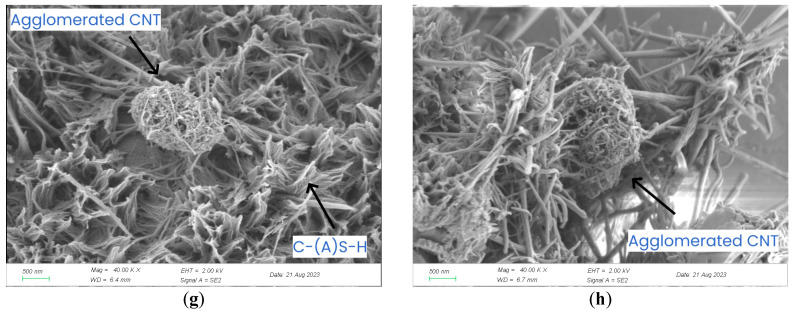
Microstructures of CLTB samples with different concentrations of CNT. (**a**–**d**) 0.25% CNTPL; (**e**–**h**) 0.25% CNTCOOH.

In order to find and observe various forms of CNTs, CLTB specimens with 0.25% CNT were used. The microstructure of the CLTB specimen was determined via SEM, and the images were analyzed to identify large tailings sand particles, hexagonal Ca(OH)_2_ platelets, and acicular trisulfide-type hydrated calcium thio aluminate (AFt) hydration products. The irregular elliptical particles and flocs are CSH and C-A-H in Figure 12a and Figure 12b, respectively. It can be observed that there are pores and cracks in CLTB, C-S-H, and C-A-H generated by the hydration reaction attached to tailings, and AFt is evenly distributed around C-S-H. Therefore, the main factors to resist deformation are the friction and cohesion between the hydration products and aggregate, as well as the strength of the hydration product network.

Figure 12d–f shows the nano-effect of carbon nanotubes. Hydration products are formed around these carbon nanotubes, which are interspersed with carbon nanotubes to form a three-dimensional interpenetrating network of stronger and denser hydration products, filling the pores, enhancing the bonding effect, and increasing the strength of CLTB. As can be seen from Figure 12g,h, CNTs that were not effectively dispersed clustered and convolved to form elliptic spherical structures, failing to exert the nanoscale properties of CNTs and forming new pore spaces.

**Figure 13:**
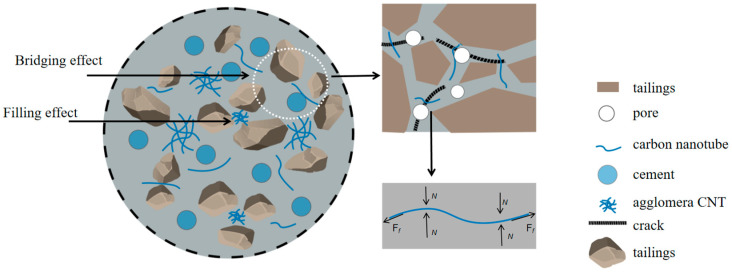
Illustration of the mechanism through which CNTs affect compressive strength. F*_f_* is the frictional force. N is the normal force.

Figure 13 shows a conceptual diagram of the CNT enhancement mechanism in CLTB, where CNT are randomly distributed in the tailing particles in their dispersed and aggregated forms. In the process of pore compaction, micro-cracks will be formed in the pores after extrusion, resulting in interfacial defects. At this point, the carbon nanotubes in the pores act as inhibitors. The friction generated by the tight bond between the carbon nanotubes and the hydrated product effectively resizes the normal force of crack development.

At the microscopic level, the nano-effect and high strength characteristics of CNTs played a role in filling pores. The three-dimensional interpenetrating network formed by nano-nucleation and bridging effectively inhibited the development of micro-pores into micro-cracks and the further development of cracks.

## 4. Conclusions

In this study, the effects of adding CNTs to CLTB specimens were studied through uniaxial compression tests, theoretical analysis, and SEM. Based on the results, the following conclusions can be drawn:

(1) CNTPL and CNTCOOH were enhanced well when the CNT content was less than 0.2%, and CNTCOOH with good dispersion enhanced samples better when the CNT content was greater than 0.2%. When the cement tailings ratio was 1:8, the UCS test strength of 0.02% CNTPL-enhanced and 0.02% cement-enhanced CLTB samples were 1.12 MPa and 1.25 MPa, respectively. Compared with ordinary CLTB samples, 0.02% CNTPL and 0.02% CLTB samples were 75% and 95.3%, respectively; these results show that the addition of appropriate CNTs can significantly increase the packing strength. The stress-change curve and damage process analysis show that CNT can only play a role at the microscopic level due to its small size, so it does not change the damage stage of CLTB but optimizes the damage process and slows down the rapid accumulation of damage values.
(2) According to FTIR and SEM, CNT has nano-nucleation, bridging, and filling to form a network skeleton of carbon nanotube hydration products, which effectively improves the mechanical properties of CLTB samples and inhibits the development of micro-cracks.

Through this experiment, it was found that both kinds of carbon nanotubes can improve the effect of cementing filler. The strengthening effects of different functional carbon nanotubes are also different, and further studies can be performed on other functional CNTs. CNTs, as a renewable, pollution-free additive, improve the tailings content in CLTB and reduce the amount of cement, making the cemented filling technology more economical and environmental. CNTs can inhibit the development of micro-cracks but cannot inhibit the development of large cracks. In the later stage, the development of large cracks can be minimized by adding an appropriate amount of green materials such as basalt fibers and waste rubber fibers.

## Figures and Tables

**Table 1:** Physical parameters of carbon nanotubes.

Type	Length (μm)	Outer Diameter (nm)	Inner Diameter (nm)	Density (g/cm^3^)	Purity (wt.%)
CNTPL	3–12	8–15	3–5	0.08	>95
CNTCOOH	2–8	10–15	5–8	0.10	>95

**Table 2:** Mix proportions of CLTB.

Samples	Cement-Tailing Ratio	CNTPL (wt.%)	CNTCOOH (wt.%)	Slurry Concentration Ratio (W)
CLTB-1	1:4			0.7
CLTB-2	1:6			0.7
CLTB-3	1:8			0.7
R-CLTB-1	1:8	0.05		0.7
R-CLTB-2	1:8	0.1		0.7
R-CLTB-3	1:8	0.15		0.7
R-CLTB-4	1:8	0.2		0.7
R-CLTB-5	1:8	0.25		0.7
R-CLTB-6	1:8		0.05	0.7
R-CLTB-7	1:8		0.1	0.7
R-CLTB-8	1:8		0.15	0.7
R-CLTB-9	1:8		0.2	0.7
R-CLTB-10	1:8		0.25	0.7

## Data Availability

Data are contained within the article.
